# Epidemiology, Symptoms and Pathophysiology of Long Covid Complications

**DOI:** 10.33696/immunology.6.209

**Published:** 2024

**Authors:** Chongyang Zhang, Chiung-Yu Hung, Chia George Hsu

**Affiliations:** 1 Department of Pharmacology and Physiology, University of Rochester, School of Medicine and Dentistry, Rochester, NY 14642, USA; 2 Department of Molecular Microbiology and Immunology, South Texas Center for Emerging Infectious Diseases, The University of Texas at San Antonio, San Antonio, TX 78249, USA; 3 Department of Kinesiology, The University of Texas at San Antonio, San Antonio, TX 78249, USA

**Keywords:** COVID-19, Long COVID complications, Epidemiology, Symptoms, Pathophysiology

## Abstract

Long COVID, or post-acute sequelae of SARS-CoV-2 infection, reports to affect a significant proportion of COVID-19 survivors, leading to persistent and multi-organ complications. This review examines the epidemiology, symptoms of long COVID complications, including cardiac, hematological, vascular, pulmonary, neuropsychiatric, renal, gastrointestinal, musculoskeletal, immune dysregulation, and dermatological issues. By synthesizing the latest research, this article provides a comprehensive overview of the prevalence and detailed pathophysiological mechanisms underlying these complications. The purpose of this review is to enhance the understanding of diverse and complex nature of long COVID and emphasize the need for ongoing research, seeking to support future studies for better management of long COVID.

## Introduction

In late 2019, a novel and potentially deadly virus causing COVID-19, later identified as SARS-CoV-2, led to severe respiratory illness and quickly spread across the globe, reaching pandemic status by early 2020. This swift transmission overwhelmed healthcare systems and led to widespread emergency measures. Recognizing the escalating crisis, the World Health Organization declared COVID-19 a pandemic on March 11, 2020. As of 9th October 2024, the virus infected over 103.8 million people and claimed more than 1.12 million lives in the United States [1].

As the pandemic progressed, long COVID (also known as post-acute sequelae of SARS-CoV-2 infection) became a pressing issue, with millions experiencing persistent and debilitating symptoms long after their initial recovery, underscoring the need for ongoing research and targeted treatments. According to World Health Organization, long COVID is defined as “the continuation or development of new symptoms 3 months after the initial SARS-CoV-2 infection, with these symptoms lasting for at least 2 months with no other explanation.” According to the Morbidity and Mortality Weekly Report from the CDC, 6.9% of U.S. adults have reported experiencing long COVID [2], while multiple large-scale or nationwide surveys suggest 14% to 47% of COVID-infected patients experience long COVID symptoms, underscoring its widespread impact [3–5]. Moreover, long COVID patients face a significantly higher risk of all-cause mortality compared to controls [6]. Without effective intervention, many individuals may develop lifelong disabilities due to the diverse multi-organ complications associated with long COVID, highlighting the enduring health implications of COVID-19.

The purpose of this review is to comprehensively analyze long COVID, with a specific emphasis on the epidemiology of its various complications, symptoms, and underlying pathophysiological mechanisms. This article aims to provide updated insights into all types of long COVID complications, offering a thorough exploration of the pathophysiological mechanisms behind. By integrating recent research findings, this review aims to deepen understanding and contribute to the development of strategies for managing long COVID.

## Cardiac Complications

Studies have revealed persistent myocardial injury in a significant proportion of COVID-19 survivors. A large retrospective study from England of 47,780 patients found a 5-fold increase in major cardiac events within 140 days post-COVID-19 compared to controls [7]. At the 6-month follow-up, cardiovascular magnetic resonance abnormalities were found in half of the patients who recovered from COVID-19 with elevated in-hospital high-sensitivity troponin levels [8]. Another study of 154,000 US veterans revealed that compared to controls, patients with long COVID syndrome at the 12-month mark have a 1.6 times higher risk of new onset cardiovascular disease of any type [9].

Post-COVID cardiac complications include angina, myocardial ischemia, myocarditis, pericarditis, elevated troponin levels, arrhythmias, atrial fibrillation and heart failure. These heightened risks were observed up to a year after the acute phase and were evident even among those who were not hospitalized. For every 1000 people, post-COVID-19 at 12 months was associated with an extra 19.86 more incidents of dysrhythmias; 7.28 more incidents of ischemic heart disease including 5.35 incidents of acute coronary disease, 2.91 incidents of myocardial infarction, and 2.5 incidents of angina; 1.23 incidents of inflammatory disease of the heart or pericardium, including 0.98 incidents of pericarditis and 0.31 incidents of myocarditis [9].

Understanding how COVID-19 causes cardiac injury reveals its complex cardiovascular implications. Fever and inflammation accelerate heart rate, increasing metabolic demands, while compromised lung function leads to oxygen supply-demand mismatch and potential heart damage [10]. Additionally, persistent chest pain in post-COVID patients often stems from coronary microvascular dysfunction, resulting in myocardial ischemia and angina-like symptoms [11,12]. Direct virus infection of cardiomyocytes triggers myocarditis, characterized by inflammatory infiltrates and non-ischemic myocardial necrosis [10]. Furthermore, the intense inflammatory response, known as the cytokine storm, releases excessive cytokines (TNF-α, IL-1, and IL-6), causing myocardial injury, vascular leakage and interstitial edema, impairing heart function [8,10]. Moreover, COVID-19 may provoke autoimmune responses targeting cardiac self-antigens through molecular mimicry or bystander loss-of-tolerance, leading to long-term cardiovascular complications including arrhythmias and myocarditis [13].

## Hematological Complications

A prothrombotic state from acute infection can persist during post-COVID recovery. A study of 154,000 US veterans found an additional 9.88 thromboembolic incidents per 1,000 individuals post-COVID-19 at 12 months, including 5.47 pulmonary embolisms and 4.18 deep vein thromboses [9]. Similarly, a cohort study of 48 million adults in England and Wales showed that while the incidence of arterial and venous thromboembolic events declines over time, it remains elevated up to 12 months post-COVID-19 compared to no COVID-19 diagnosis control [14]. Post-COVID hematological complications include microvascular, venous, and arterial thrombosis.

SARS-CoV-2 infection can disrupt the endothelium, leading to a procoagulant and hypofibrinolytic state. This is characterized by activated endothelial cells-either directly infected by the virus or stimulated by a cytokine storm-producing increased amounts of von Willebrand factor and factor VIII, which promote platelet adhesion and aggregation and thus induce clot formation [15]. Additionally, endothelial cells generate higher levels of plasminogen activator inhibitor-1, which inhibits clot degradation, resulting in a hypofibrinolytic state [16]. COVID-19 infection and inflammatory stimuli also induce tissue factor expression in monocytes and macrophages, releasing tissue factor into the bloodstream, where it forms complexes with factor VIIa, leading to extensive platelet activation and coagulation [17–19]. Furthermore, SARS-CoV-2 can directly infect neutrophils, causing the production of neutrophil extracellular traps that activate factor XII, triggering the intrinsic pathway of the coagulation cascade [10].

## Vascular Complications

Vascular complication is an important component of long-COVID. In a cross-sectional multicenter observational study involving 618 patients with long COVID-19 symptoms, within six months post-infection 397 (49.7%) exhibited an impaired endothelial quality index, indicating endothelial dysfunction [20]. This is evidenced by other cohort studies revealing similar results that significantly higher brachial artery flow-mediated dilation — another marker of endothelial dysfunction — was observed six months post-COVID hospital discharge compared to controls [21–23]. Meanwhile, elevated levels of circulating endothelial cells, a biomarker of vascular injury, along with increased levels of vascular transformation biomarkers (Angiopoietin-1, P-selectin, Endothelin-1), were found in COVID-19 survivors one to six months post-infection compared to healthy controls [24–26]. Additional vascular complications in long COVID patients include capillary rarefaction and arterial stiffness, observed even one year after infection, evidenced by a significant decrease in vascular density in sublingual microvessels and increased carotid-femoral pulse wave velocity (a marker of arterial stiffness) [27,28].

The vascular endotheliopathy following SARS-CoV-2 infection is attributed to both direct infection and indirect damage, primarily caused by endothelial inflammation, imbalance in the renin-angiotensin-aldosterone system, and oxidative stress. The SARS-CoV-2 genome encodes 29 proteins that, when cloned and overexpressed, significantly decrease endothelial permeability tight junction proteins and increase von Willebrand factor expression and interleukin-6 (IL-6) levels, indicating endothelial dysfunction [29]. Direct infection by SARS-CoV-2 induces endothelial inflammation and permeability through integrin α5β1 and NF-κB signaling, which in turn stimulate the expression of leukocyte adhesion molecules (VCAM1 and ICAM1), coagulation factors (TF and FVIII), proinflammatory cytokines (TNF-α, IL-1β, and IL-6), as well as the adhesion of peripheral blood leukocytes and hyperpermeability of the endothelial cell monolayer, both *in vitro* and *in vivo* [30]. Indirect endothelial inflammation can also result from cytokine storms or leukocyte adhesion. In COVID-19 patients, the cytokine storm exposes the endothelium to pro-inflammatory cytokines such as IL-6, interleukin-1β (IL-1β), and tumor necrosis factor-α (TNF-α). Binding of these cytokines to their receptors on endothelial cells increases vascular permeability, induces capillary leakage, and amplifies the cytokine storm by further increasing the secretion of IL-6, IL-8, and MCP-1 by endothelial cells [15]. Proinflammatory cytokines also elevate the expression of leukocyte adhesion molecules on endothelial cells, including E-selectin, P-selectin, and L-selectin, facilitating the interaction and recruitment of various classes of blood leukocytes in response to innate and adaptive immunity [16].

The renin-angiotensin-aldosterone system (RAAS) is essential for maintaining vascular tone. ACE2, the main receptor for SARS-CoV-2, is found on the vascular endothelium and counter-regulates the RAAS pathway. SARS-CoV-2 binding to ACE2 reduces ACE2 levels, leading to less Ang II inactivation and decreased conversion to angiotensin-(1–7). Accumulated Ang II causes vasoconstriction, endothelial activation, and production of IL-6 as well as reactive oxygen species through the AT1 receptor [31,32]. Endothelial cell activation and regulation of adhesion molecules also trigger neutrophil activation, producing numerous histotoxic mediators including reactive oxygen species, that disrupt antioxidant mechanisms like Nrf2 and increase vascular permeability and leukocyte adhesion [17].

## Pulmonary Complications

COVID-19 presents diverse clinical symptoms, predominantly respiratory in nature. While the exact epidemiology of COVID-19’s pulmonary vascular effects remains unclear, studies suggest a significant involvement of the lungs and pulmonary vasculature. Host factors such as age, sex, and pre-existing conditions influence disease severity, highlighting the complexity of its epidemiological patterns. Notably, pulmonary hypertension and pulmonary artery hypertension are observed in a subset of patients, characterized by elevated pressure in the pulmonary circulation [33]. Lung fibrosis, a common sequela of severe COVID-19, involves the deposition of excess collagen and extracellular matrix, leading to irreversible structural changes and impaired lung function [34]. Dyspnea, a hallmark symptom, manifests as shortness of breath and is often accompanied by ventilator dependence and oxygen dependence in severe cases [35]. These respiratory complications significantly impair quality of life and may persist beyond the acute phase of infection, necessitating comprehensive post-acute care and rehabilitation programs tailored to address respiratory sequelae.

The pathophysiology of COVID-19-related lung injury involves intricate cellular and molecular processes. Upon infection, SARS-CoV-2 directly targets alveolar epithelial cells, leading to cell death and disruption of the lung barrier function [36]. This triggers an inflammatory cascade characterized by the release of pro-inflammatory cytokines, recruitment of immune cells such as lymphocytes and macrophages, and activation of fibroblasts, which contribute to tissue fibrosis [37]. Endothelial dysfunction and microthrombi formation further compromise pulmonary vascular integrity, aggravating lung injury. Hypoxia pathways play a pivotal role, exacerbating endothelial dysfunction and cytokine storms, while complement activation amplifies inflammation and coagulation abnormalities [38,39]. Recent studies highlight ferroptosis, an iron-dependent form of cell death, as a key driver of COVID-19-related lung damage [40,41]. This discovery suggests that targeting ferroptosis with specific therapies could improve outcomes in severe cases of COVID-19. Ferroptosis involves oxidative damage that causes the collapse of cell membranes. Evidence links ferroptosis to severe COVID-19 lung pathology, with patients showing signs of hyperferritinemia and disrupted iron regulation. Autopsy analyses reveal elevated markers of ferroptosis, such as transferrin receptor 1 and malondialdehyde, correlating with severe lung injury. Iron overload in experimental models promotes ferroptosis in lung cells, further supporting its role in COVID-19 lung damage [41]. These findings suggest that inhibiting ferroptosis could offer a promising therapeutic approach to reduce lung injury in SARS-CoV-2 infection. Understanding these complex mechanisms is essential for developing targeted therapeutic strategies to mitigate lung damage and improve outcomes in severe COVID-19 cases.

## Neuropsychiatric Complications

Neuropsychiatric symptoms in COVID-19 survivors vary in incidence across studies. A study of 154,000 US veterans reported 5.48 additional cases of cerebrovascular disorders, including 4.03 strokes, per 1,000 individuals within 12 months post-COVID-19 [9]. Follow-up studies in Germany and the UK found that 20% to 70% of patients, including young adults, experienced neuropsychiatric symptoms months after respiratory symptoms resolved, indicating persistent brain involvement [42]. Multiple retrospective cohort studies also found that one-third of COVID-19 survivors developed neurological or psychiatric symptoms six months after infection [43]. These symptoms include sleep difficulties, fatigue, headache, loss of smell and taste, cognitive and attention deficits (brain fog), new-onset anxiety, depression, and psychosis [42].

Several mechanisms contribute to the neuropsychiatric symptoms observed in COVID-19 survivors. SARS-CoV-2 invades brain capillary endothelial cells via the ACE2 receptor, facilitated by the enzyme TMPRSS2 [43]. The virus can also cross the blood-brain barrier (BBB) due to cytokine-induced BBB instability or through monocytes [44]. Inflammatory cytokines (e.g., IL-1β, IL-10, IL-6, TNF-α) are elevated in cerebrospinal fluid during a cytokine storm, activating local microglia and astrocytes [45]. This leads to increased production of kynurenine, quinolinic acid, glutamate, and upregulation of NMDA receptors, as well as depletion of neurotransmitters such as serotonin, dopamine, and norepinephrine [46]. Additionally, the coagulation cascade and elevated von Willebrand factor result in thrombotic events [47]. Altered neurotransmission, excitotoxicity from increased glutamate, and hypoxic injury further contribute to neuronal dysfunction and loss [48]. The specific neuropsychiatric symptoms vary depending on the affected Brodmann area.

## Renal Complications

People who have recovered from COVID-19 face a higher risk of kidney disease, even if their symptoms were mild to moderate and did not require hospitalization. Data from the Veterans Health Administration in the US, involving 89,216 COVID-19 survivors (at least 30 days post-recovery) and 1,637,467 non-infected controls, shows that COVID-19 survivors have an increased risk of annual eGFR decline, acute kidney injury, major adverse kidney events, and end-stage kidney disease [49,50]. Kidney disease in COVID-19 patients often begins as acute kidney injury and may progress to chronic kidney disease in survivors [51]. Other renal issues in long COVID include tubulointerstitial fibrosis, tubular necrosis, glomerulopathy, microvascular thrombi, hematuria, and proteinuria [52].

Several pathophysiological mechanisms contribute to renal complications in long COVID, including direct viral entry, systemic hypoxia, inflammatory cytokines, and abnormal coagulation. Kidney cells possess ACE2 receptors that enable SARS-CoV-2 direct infection. Studies with kidney organoids devoid of immune cells have shown that direct infection by SARS-CoV-2 can injure renal cells and activate profibrotic signaling pathways, impairing kidney function and potentially leading to chronic kidney disease [53]. Inflammatory cytokines from a cytokine storm can damage the endothelial cells lining kidney blood vessels, increasing vascular permeability, causing edema, and reducing blood flow, which results in tissue hypoxia and kidney injury [54]. Cytokines can also directly harm renal cells, including tubular epithelial cells and podocytes, disrupting their normal function and causing acute tubular necrosis and glomerular injury. Additionally, cytokine storms can trigger the coagulation cascade, leading to microvascular thrombosis [55,56]. These small blood clots can obstruct the tiny blood vessels in the kidneys, causing ischemia and infarction of renal tissue.

## Gastrointestinal Complications

Gastrointestinal complications are a notable aspect of long COVID, affecting a significant portion of patients. Various studies have reported 16–44% of patients experiencing gastrointestinal symptoms by 100 days post-COVID infection [57–59], while COVID survivors reported significantly higher rates of irritable bowel syndrome even 1 year after infection versus controls [60]. More broadly, a meta-analysis of 50 studies found that 22% of patients experienced gastrointestinal symptoms as part of long COVID, including nausea, vomiting, loss of appetite, dyspepsia, irritable bowel syndrome, diarrhea, abdominal pain, constipation, highlighting the persistent and diverse nature of gastrointestinal issues following COVID-19 [61].

Gastrointestinal complications in long COVID can be attributed to three primary mechanisms: direct viral infection, alterations in gut-brain signaling, and disruption of the normal intestinal microbiome. SARS-CoV-2 is known to infect the epithelial lining of the gastrointestinal tract, liver cells, and bile ducts via ACE2 receptors, causing direct damage to these organs [60]. Additionally, the virus can induce persistent intestinal immune abnormalities, characterized by an enrichment of cytotoxic T cells and unique T cell dynamics, contributing to gastrointestinal post-acute COVID-19 syndrome [62]. Alterations in gut-brain signaling are also significant, as this complex network of nerves controls various digestive functions. Disruptions in communication between the brain and the gut, known as disordered gut-brain interactions, may lead to prolonged gastrointestinal distress in long COVID patients [63]. Lastly, infection-induced general inflammation can disrupt the normal gut microbiota, reducing microbial diversity and altering specific gut microbiome profiles, which are essential for maintaining overall health. These changes have been observed in studies showing a correlation between reduced microbial diversity and the prevalence of long COVID symptoms [64].

## Musculoskeletal Complications

Musculoskeletal complications are a major and common consequence of long COVID, impacting many survivors. Studies indicate that persistent musculoskeletal pain is prevalent in COVID-19 survivors months after the acute phase of the illness. A large retrospective study involving 273,618 COVID survivors found that within 180 days of acute infection, 3% reported myalgia, and 12% had other types of pain [65]. Among hospitalized COVID-19 patients, other studies show that 47–53% reported persistent muscle pain and joint pain three to six months after hospital discharge [66–68]. Even in non-hospitalized patients with mild COVID-19, 71% experienced persistent muscle pain and 44% reported joint pain one to two months after acute illness [69]. These findings highlight the pervasive and enduring nature of musculoskeletal complications in long COVID. Musculoskeletal symptoms in long COVID can include widespread pain in muscles and joints throughout the body, manifesting as chest tightness, muscle aches, joint pain, stiff neck, muscle spasms, and bone aches or burning sensations. Additionally, patients may experience excessive fatigue, myalgia, arthralgia, muscle weakness, and skeletal muscle damage.

Musculoskeletal complications in long COVID can be attributed to direct viral infection, systemic inflammatory responses, and lifestyle changes. Studies have shown the expression of ACE2 receptors and TMPRSS2 proteins in skeletal muscle, smooth muscle, cartilage, synovia, and cortical bone, indicating that SARS-CoV-2 can directly infect these tissues. This infection can impair muscle structure and lower exercise capacity [70]. Additionally, systemic inflammatory responses in long COVID patients, marked by elevated interferon gamma, C-reactive protein, interleukins (IL-6, IL-2, IL-10), and tumor necrosis factor alpha, can impair muscle function and endurance by disrupting muscle protein metabolism through triggering catabolic pathways and suppressing anabolism [71]. Furthermore, changes in lifestyle during and post the pandemic, such as altered work and sleep patterns may also contribute to the decline in musculoskeletal function and conditions [72].

## Immune Dysregulation Complications

Inflammatory cytokines and immune dysregulation remain elevated from the acute COVID infection phase in long COVID patients. Multiple studies have demonstrated this by examining the profiles of inflammatory proteins in patients’ plasma months after COVID infection [73]. For instance, a study that analyzed 368 immune mediators in the blood plasma of 428 long COVID patients with various persistent symptoms lasting at least three months found markers indicative of myeloid inflammation, such as interleukin-1 receptor 2 and matrillin-2, as well as markers of complement activation, such as collectin-12 and C1QA, consistently associated with all long COVID symptoms [74]. Another study using proteomics measured over 6,500 proteins in the serum of long COVID patients and found that increased complement activation persisted beyond the acute phase and remained at the six-month follow-up, underscoring the prolonged immune system activation in long COVID patients [75].

Long COVID significantly impacts the innate immune system in terms of how neutrophils, monocytes, and macrophages behave. Studies reveal that while neutrophil extracellular traps are primarily formed during acute infection, their formation persists abnormally in long COVID patients [76]. This persistent activation might be due to epigenetic changes in long-lived parenchymal and immune cells or reprogramming in the bone marrow, leading newly differentiated neutrophils to become excessively activated [77]. Monocytes also exhibit prolonged activation in long COVID, with higher frequencies of intermediate (CD14^+^ CD16^+^) and non-classical (CD14^−^ CD16^+^) monocytes observed up to 15 months post-infection [78]. This prolonged activation might stem from interactions between hematopoietic progenitor cells and T cells, causing long-lasting changes in the monocyte compartment. Furthermore, macrophages show an enduring inflammatory response in long COVID. Tissue-resident macrophages, which are crucial for immune defense and tissue homeostasis, exhibit an inflammatory transcriptional and metabolic imprint lasting for months post-infection [79–81]. These macrophages, found in various tissues like the brain, liver, lungs, and adipose tissue, continue to produce pro-inflammatory eicosanoids and downregulate pro-resolving factors, potentially driving the chronic inflammation seen in long COVID.

An emerging question is how immune cells, particularly macrophages, are able to withstand prolonged oxidative stress in the damaged lung following infection. Recent studies have identified that inflammatory macrophages exhibit heightened resistance to ferroptosis, a form of programmed cell death characterized by lipid peroxidation [82]. Several mechanisms contribute to this resistance: macrophages rely on glycolytic metabolism to increase nicotinamide adenine dinucleotide phosphate (NADPH) production, which plays a critical role in regulating reactive oxygen species (ROS) and facilitating glutathione synthesis [83,84]. Additionally, macrophages produce nitric oxide, which mitigates lipid peroxidation, while the activation of nuclear factor erythroid 2-related factor 2 (NRF2) in response to oxidative stress further enhances antioxidant defense pathways [80,85]. Moreover, macrophages may exhibit altered lipid profiles, including lower levels of polyunsaturated fatty acids (PUFAs), which may contribute to their enhanced resistance to ferroptosis [86–88]. Understanding these adaptive mechanisms is crucial for elucidating the role of ferroptosis in macrophages and its contribution to post-COVID pathophysiology and dysregulated immune responses.

Besides innate immune system, the adaptive immune system also shows significant alterations of T cells and B cells in long COVID. Studies indicate that individuals with long COVID exhibit lower and more rapidly waning nucleocapsid-specific cytotoxic CD8^+^ T cells compared to those without long COVID [89]. Additionally, patients with long COVID have reduced numbers of CD4^+^ and CD8^+^ effector memory cells and increased PD-1 expression on central memory cells, which may suggest decreased functional capacity or persistent antigenic stimulation [90]. Contrarily, some research reports higher frequencies of interferon-gamma (IFN-γ) and TNF-producing SARS-CoV-2-specific T cells in long COVID patients, along with elevated plasma inflammatory biomarkers (IL-6 and CRP), correlating with increased immune activation and the presence of long COVID [91]. Dysregulated tissue-resident memory CD8^+^ T cell responses in the lungs of long COVID patients have also been associated with impaired lung function, potentially due to the persistence of viral antigens [92]. Regarding B cells, studies have identified the presence of B cell-derived autoantibodies in long COVID pathogenesis. A transient, naive-derived antibody-secreting B cell compartment, enriched in autoreactive potential, emerges during the acute phase of severe COVID-19 [93]. These cells typically regress during recovery, but in some long COVID patients, autoantibodies persist, suggesting a potential role in the ongoing pathogenesis of long COVID.

Autoantibodies, cytokine storms, and residual viral components also significantly contribute to the dysregulated immune system in long COVID. Elevated levels of autoantibodies in long COVID target various tissues and organs, including ACE2, β2-adrenoceptor, and muscarinic M2 receptor, potentially leading to an autoimmune response that persists after viral clearance [94,95]. This autoimmune activity may cause ongoing tissue damage and inflammation, contributing to long COVID symptoms. Cytokine storms, characterized by excessive production of proinflammatory cytokines like IL-1β, IL-6, and TNF-α, disrupt normal endothelial functions, leading to thrombosis and local tissue injury [16]. Inappropriate or excessive cytokine release perturbs the protective functions of the endothelium, exacerbating pathological processes and potentially contributing to the chronic symptoms observed in long COVID patients. Residual SARS-CoV-2 viral proteins or RNA in various body tissues and fluids, such as the reproductive system, cardiovascular system, brain, and plasma, have been identified in several studies [96,97]. This persistent viral presence suggests active reservoirs of the virus or its components, which may continuously stimulate the immune system and contribute to ongoing inflammation and symptoms. For instance, SARS-CoV-2 spike antigen was found in 60% of long COVID patients up to 12 months post-diagnosis, indicating potential viral persistence and its role in long COVID pathogenesis [98]. These mechanisms may contribute to the wide range of long COVID symptoms across multiple organ systems.

## Dermatological Complications

The frequency of skin lesions associated with COVID-19 infection varies across different studies, ranging from 4% to 20.4%. For instance, a Chinese study of 1,099 positive cases reported a low incidence of only 0.2% [99]. In contrast, an Italian series of 88 patients documented a much higher incidence of 20.4% [100], while a study in Europe found a rate of 6.6% among 1,293 patients [101]. Additionally, in a Cross-Sectional Study conducted at Jordan Hospital, 7.55% of 821 patients developed rashes [102]. Skin manifestations associated with SARS-CoV-2 infection can be categorized into five primary groups: Chilblain-like lesions, maculopapular eruptions, urticarial eruptions, vesicular eruptions, and livedo or necrosis [102,103]. Additionally, cases of pityriasis rosea [104] and shingles [105] have also been documented. Interestingly, nearly half of these skin findings occurred simultaneously with the onset of other COVID-19 symptoms (46.1% of all cases). For the remaining cases, rashes either appeared shortly after (44.3%) or before (9.6%) the non-cutaneous COVID-19 manifestations [106].

COVID-19-induced skin manifestations arise through two known mechanisms: inflammation and vascular damage. Certain skin cells, such as keratinocytes (found in the outermost skin layer), eccrine gland epithelial cells (lining the sweat gland tube), and the basal layer of hair follicles express ACE2 receptors [107]. Direct infection of these skin cells by SARS-CoV-2 via ACE2 can lead to skin damage. Furthermore, the elevated levels of inflammatory cytokines triggered by COVID-19 contribute to perivascular infiltration of inflammatory cells, vasodilation, and edema, ultimately leading to inflammation-induced skin eruptions. Such eruptions include maculopapular/morbilliform rashes, urticarial rashes, and vesicular rashes [108]. Vascular skin eruptions occur due to the SARS-CoV-2 infection of endothelial cells in subcutaneous capillaries and veins though ACE2 receptors. This can result in endothelial dysfunction and microthrombosis within the skin, and manifest as chilblain-like rashes, petechiae/purpura, and livedo reticularis [109].

## Conclusion

**Figure 1.**
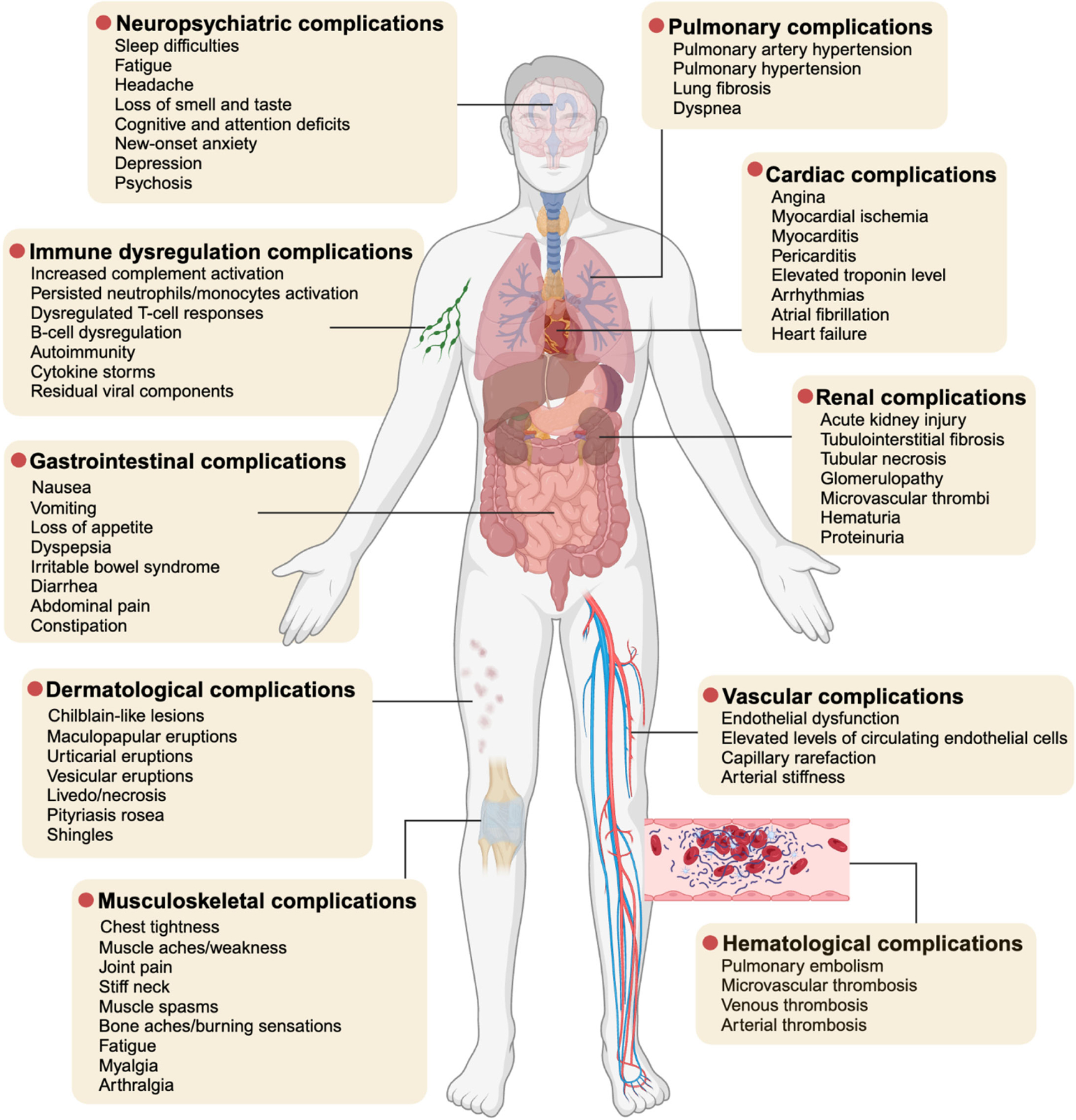
Symptoms Associated with Long COVID Complications. This figure illustrates the common symptoms experienced by patients with various types of long COVID complications reported in different organ systems. (Figure created in BioRender.com).

Long COVID presents a multisystemic illness impacting multiple organ systems, including respiratory, cardiovascular, neurological, and gastrointestinal systems, along with vascular and clotting abnormalities (Figure 1). It has debilitated millions worldwide, with numbers continuing to grow. Epidemiological data and over 4 years of research indicate that without action, many may face lifelong disabilities. Diagnostic and treatment options are currently insufficient. Recent studies highlight the significant accumulation of ferroptosis markers, such as 4-hydroxynonenal, transferrin receptor 1, and malondialdehyde, in fatal cases. Future research should focus on measuring these ferroptosis biomarkers using lipidomic analysis to enhance early detection and monitoring of long COVID [38,110]. Urgent clinical trials are needed to test treatments targeting underlying biological mechanisms such as viral persistence, neuroinflammation, excessive blood clotting, and autoimmunity. As of now, ClinicalTrials.gov lists 542 related trials, with only 118 categorized as interventional and recruiting, and just 20 focused on pharmacological treatments. The remaining studies cover various supportive therapies like rehabilitation, telehealth, and alternative interventions, highlighting a gap in therapies targeting core pathophysiology. Given the profound impact of long COVID on individuals and economies, the pace and scope of current clinical trials need acceleration. Therefore, addressing the pathophysiological mechanisms of long COVID is crucial for improving patient outcomes and developing comprehensive management strategies.
